# A New Synergetic Nanocomposite for Dye Degradation in Dark and Light

**DOI:** 10.1038/srep38606

**Published:** 2016-12-08

**Authors:** Lakshmi Prasanna V., Vijayaraghavan Rajagopalan

**Affiliations:** 1 Department of Chemistry, School of Advanced Sciences, VIT University, 632 014 -Vellore, India

## Abstract

Environmental hazard caused due to the release of dyes in effluents is a concern in many countries. Among the various methods to combat this problem, Advanced Oxidation Process, in which semiconductor photocatalysts are used, is considered the most effective one. These materials release Reactive Oxygen Species (ROS) such as hydroxyl radical and superoxide in suspension that degrade the dyes into non-toxic minerals. However, this process requires visible or UV light for activation. Hence, there is a need to develop materials that release ROS, both in the absence and in the presence of light, so that the efficiency of dye removal is enhanced. Towards this objective, we have designed and synthesized a new nanocomposite ZnO_2_/polypyrrole which releases ROS even in the dark. The ROS released in the dark and in light were estimated by standard methods. It is to be noted that ZnO_2_ degrades the dye only under UV light but not in dark or in the presence of visible light. We propose the mechanism of dye degradation in dark and light. The synergically coupled nanocomposite of ZnO_2_/ppy is the first example that degrades dyes in the dark, through advanced oxidation process without employing additional reagents.

Organic dyes, which are extensively used in industries such as textile, leather, paint, printing inks, plastics, food, drugs and cosmetics, are released into water bodies, resulting in high Chemical Oxygen Demand (COD) even after being treated^1^. Among these, textile industries release the highest amount of non-biodegradable dye effluents into the environment. This discharge is a serious concern to humans and aquatic ecosystem^2^,^3^,^4^. Dyes containing N group such as Rhodamine B (RhB) and Methylene Blue (MB) are resistant to photolysis and these undergo reductive anaerobic degradation resulting in carcinogenic products^5^. Hence, the removal of these dyes from effluents through degradation into non-toxic components is of much importance to resolve the environmental problem. Conventional effluent treatment methods include precipitation, chemical oxidation, coagulation- flocculation, adsorption, filtration and reverse osmosis^6^,^7^,^8^. These methods require additional treatment and are not cost effective.

Although biological treatment is found to be effective in controlling COD and BOD, it is ineffective for complete degradation of many textile dyes^9^. Coagulation is able to degrade insoluble dyes but it is ineffective for soluble dyes^9^. Adsorption method converts these dyes from one form to another. Recently, Advanced Oxidation Processes (AOP) such as Fenton, photo Fenton, ozonisation, semiconductor based photocatalysis, photolysis using H_2_O_2_ have been found to be promising methods for dye degradation. In particular, photocatalysis carried out in ambient conditions, using semiconductor materials is gaining importance, as indicated by the volume of basic and applied research carried out in the field. Fenton catalysts have also been studied extensively for their ability to degrade dyes in the presence of UV and visible irradiation^10^,^11^. When H_2_O_2_ is added to the catalyst based on iron, hydroxyl radicals are produced which degrades toxic dyes to non-toxic components^11^. The advantages of Fenton degradation are its simplicity and the ability to work even in the absence of irradiation while its disadvantages include storage and transportation of H_2_O_2_. Further, it works only in acidic medium, and hence the method is not feasible for industrial applications^12^.

In general, wide band gap semi-conductors have been identified as heterogeneous photocatalysts and exhibit high redox potential of photogenerated charge carriers^13^. During the process, there is an excitation of electrons from the filled valence band (VB) to the empty conduction band (CB) of the semiconductors when irradiated with light of energy equal to or greater than the band gap (E_g_) of the semi-conductor. These photogenerated charge carriers through redox reactions with water, oxygen results in highly Reactive Oxygen Species (‘OH, O_2_^−^,). These ROS rapidly degrade the dyes or organic pollutants present in the medium into CO_2_ and minerals^13^. Prevention of recombination of photocharge carriers is a crucial factor in determining the quantum efficiency of the process. The size, shape, surface defects, surface functional groups and crytsallinity determine the photocatalytic property of nanomaterials^14^. A smaller size with high surface area exposes more active sites of nanomaterials, thereby enhancing the catalytic efficiency^15^. One dimensional nanomaterials like rods and wires, due to their dimensional anisotropy, have more active sites on their surface for trapping electrons and holes^14^. Annealing, structural directing agents have been tried to tune the aspect ratio of nanomaterials in order to enhance the availability of active sites^14^,^16^. Surface oxygen vacancies which act as electron traps also play a significant role in enhancing the photoctalystic activity^17^. The presence of ions such as hydroxyl, phosphate on the surface of nanomaterials increase photocatatlytic efficiency. Hydroxyl ions can adsorb on holes resulting in hydroxyl radical, while phosphate ions adsorb on holes, preventing the recombination^18^,^19^.

Degussa P 25, a mixed compound with both anatase and rutile TiO_2_ is widely used as commercialized photocatalyst^20^. Many research groups have attempted to reduce the band gap of wide band gap semi-conductors from UV-region to visible region in order to harness maximum region of solar spectrum. Such attempts include doping/co-doping with metal/non-metal ions^21^,^22^,^23^,^24^, compositing with activated carbon/CNT/fullerenes/graphene^25^,^26^ and coupling with narrow band gap semiconductors^27^,^28^,^29^. It has been established that dopants act as trapping sites for photocharge carriers, lowering the efficiency. Another beneficial approach is to couple conductive polymers (CP) with the wide band gap semi-conductors because the former, due its π conjugated electron systems, not only act as photosensitizer but these can also inject electrons into the conduction band of semi-conductors of appropriate band structure. Some polymers are photocatalysts, which are as good as TiO_2_ - P25 itself^30^,^31^. Among the nanocomposites of semiconductors, TiO_2_ modification with polyaniline^32^,^33^ and TiO_2_ with polypyrrole^34^,^35^,^36^,^37^ are important since these nanocomposites are found to be better than TiO_2_ itself in the degradation of dyes and these work under sun light as well. However, these cannot degrade dyes in dark.

We considered it worthwhile to develop a nanocomposite that generates ROS, both in the presence and absence of light, without using additional reagents, as it can significantly enhance the efficiency of dye degradation. Indeed, we could succeed in designing a new nanocomposite derived from ZnO_2_and polypyrrole that could degrade dyes such as Rhodamine B and Methylene Blue significantly, both in the absence and presence of light. We report the synthesis, characterization and dye degradation studies along with the mechanism of ROS generation in dark and in visible & UV-light. It is to be noted that neither ZnO_2_ nor polypyrrole could degrade these dyes in the presence or absence of visible light. The physio chemical synergy tuned between ZnO_2_ and polypyrrole in the nanocomposite makes it the first example of a versatile and efficient catalyst that works in the absence and presence of light for the dye degradation through ROS formation without additional reagents. The free electrons of ppy polymer and properties of ZnO_2_ were explored for the degradation of dyes in the present work.

## Results and Discussion

### Nanocomposite synthesis

Synthesis was carried out by a modified procedure adopted for TiO_2_/ppy composite^34^. Neat ZnO_2_ is creamy white in colour. ZnO_2_ catalytically oxidizes pyrrole into ppy. Cl^−^ from HCl acts as dopant to form oxidized polypyrrole on surfaces of ZnO_2_ and the nanocomposite is brown in colour (Supplementary Fig. S1). In the absence of ZnO_2_, polymerization of pyrrole was not observed (Supplementary Fig. S1) suggesting peroxide catalyst enables *in-situ* polymerization of pyrrole. Pyrrole undergoes polymerization at 80 °C in presence of ZnO_2_. ZnO_2_ catalysed synthesis of polypyrrole is reported here for the first time.

### Characterization

**Figure 1:**
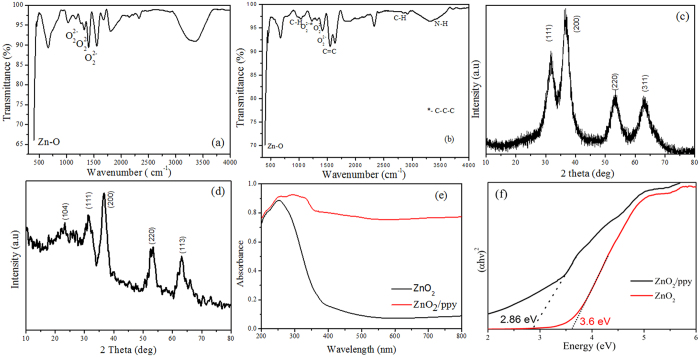
(**a**) FTIR of ZnO_2_ (**b**) FTIR of ZnO_2_/ppy (**c**) XRD of ZnO_2_(**d**) XRD of ZnO_2_/ppy (**e**) UV- DRS (**f**) Tauc plot of ZnO_2_ and ZnO_2_/ppy.

**Figure 2:**
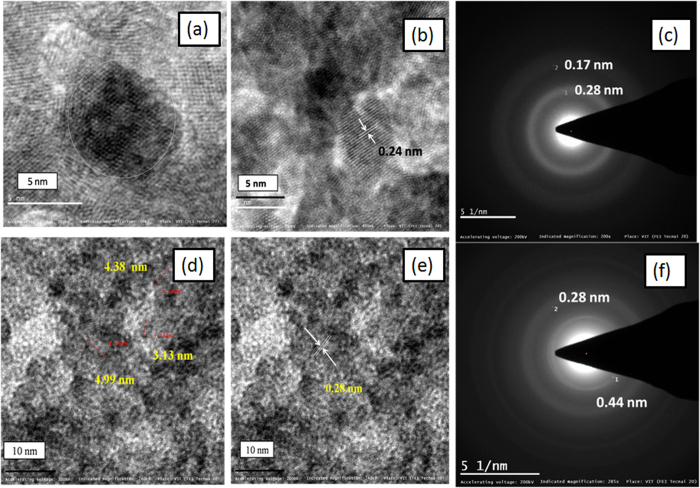
HRTEM of (**a**) and (**b**) ZnO_2_ (**c**) SAED of ZnO_2_ (**d**) and (**e**) ZnO_2_/ppy (**f**) SAED of ZnO_2_/ppy.

**Figure 3:**
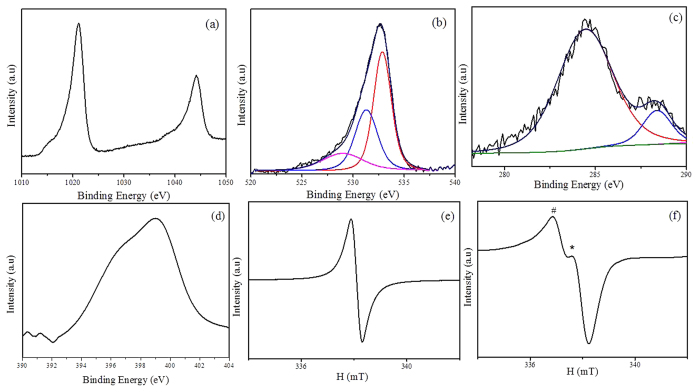
XPS of ZnO2/ppy (**a**) Zn 2p (**b**) O1s (**c**) C 1 s (**d**) N 1 s(**e**) EPR of ppy and (**f**) EPR of ZnO_2_/ppy.

Figure 1a,b show FTIR of ZnO_2_ and ZnO_2_/ppy. The peaks at 1040 cm^−1^, 1331 cm^−1^ and 1420 cm^−1^ in both products correspond to O_2_^2−^ species and 410 cm^−1^ corresponds to Zn-O bond^38^. The peaks at 3430, 2862, 1566, 1236, 965 cm^−1^ correspond to N-H, C-H, C=C, C-C-C and C-H sterching cofinfirming the presence of polypyrrole^39^. Figure 1c,d confirm single phasic cubic nanocrystalline ZnO_2_ product (JCPDS card No. 13–0311) with lattice parameter a = 4.78 Å. Figure 1d shows a characteristic peak at 24° confirming the presence of polypyrrole in ZnO_2_/ppy composite^40^. The crystallite size calculated by Scherer formula.


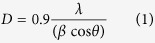


where β is the measured FWHM (in radians), θ is the Bragg angle of the peak, λ is the wavelength of X-rays. The crystallite sizes of ZnO_2_ and the composite were found to be 2.5 nm and 1.9 nm repectively. Figure 1e shows UV-DRS spectra of ZnO_2_ and ZnO_2_/ppy. ZnO_2_ shows only UV absorption at 254 nm^41^ whereas the composite shows significant absorption in visible region (400–800 nm) also as polypyrrole is expected to have absorption in visible region^42^. The optical band gap is determined from absorption spectrum using Tauc’s plot.


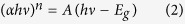


where *α* denotes absorption coefficient, *hν* is the discrete photon energy, *A* is constant, *E*_g_ is the band gap and exponent *n* depends on type of transition. Figure 1f shows band gap of ZnO_2_ and ZnO_2_/ppy. The band gap of ZnO_2_ is 3.6 eV whereas it is 2.86 eV in the composite. Figure 2 shows morphology of ZnO_2_ and ZnO_2_/ppy. ZnO_2_ is agglomerated (Supplementary Fig. S2a) with nearly spherical particles of size ~5 nm (Fig. 2a). Figure 2b shows lattice fringes of 0.24 nm confirming ZnO_2_. SAED (Fig. 3c) shows two rings with d spacing 0.26 and 0.16 nm corresponding to ZnO_2_ phase. ZnO_2_/ppy (Supplementary Fig. S2b) is dispersed with spherical particles of size below 5 nm (Fig. 2d). Figure 2e shows lattice fringe with d value 0.28 nm corresponding to ZnO_2_. SAED (Fig. 2f) shows two rings with d spacing 0.44 nm corresponding to polypyrrole^43^ and 0.28 nm corresponds to ZnO_2_. EDX shows the presence of C and N confirming the presence of polypyrrole (Supplementary Fig. S3). Figure 3a,b show Zn 2p and O 1 s XPS of ZnO_2_/ppy respectively. Zn 2p_3/2_ peak is at 1021 eV confirming Zn^2+^ valence in both the compounds^44^. O1s (Fig. 3b) shows three peaks, the peak at higher biniding energy 532.5 eV corresponds to O_2_^2−^ species and 531.2 eVcorresponds to hydroxyl species which are associated with oxygen vacancy. Peak at lower binding energy 528.4 eV corresponds to loosely bound oxygen species^45^,^46^,^47^. The presence of polypyrrole in ZnO_2_/ppy is confirmed by C1s and N 1 s (Fig. 3c,d). C 1 s shows peaks at 284.7 eV and 288.3 eV corresponding to presence of Cin α position of polypyrrole and C-O repectively.

EPR of ZnO_2_ at rooom temperature in the absence of light showed a single signal with field centre at 337.24 mT corresponding to defects (Supplementary Fig. S4)^48^. PPy showed a narrow signal with field center at 338.06 mT with g value 2.003 (Fig. 3e) confirming presence of free electrons on polymer chain^49^. ZnO_2_/ppy shows two signals with field centre at 338.09 mT. The signal which is represented by # is due to defects of ZnO_2_ and * is due to free electrons present on polymer chain of polypyrrole (Fig. 3f).

### Reactive Oxygen Species

**Figure 4:**
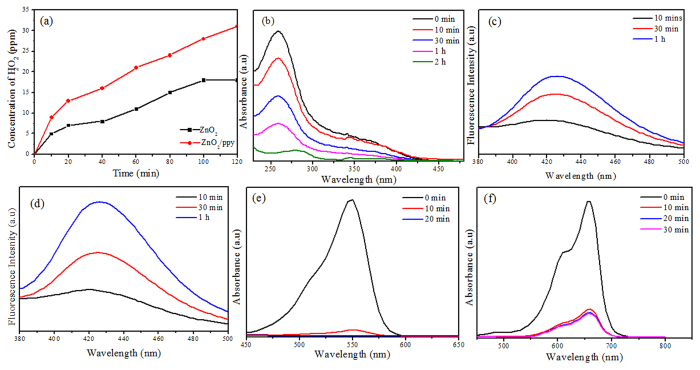
(**a**) H_2_O_2_ produced form aqueous suspension of ZnO_2_ and ZnO_2_/ppy in dark (**b**) NBT degradation by ZnO_2_/ppy in dark for 2 h (**c**) Fluorescence spectra of hydroxyl terepthalic acid from aqueous suspensions of ZnO_2_/ppy in dark (**d**) Under visible irradiation (**e**) Degradation of RhB (10 ppm) in dark by ZnO_2_/ppy in dark (**f**) Degradation of MB (5 ppm) by ZnO_2_/ppy in dark.

Figure 4a shows kinetics of H_2_O_2_ production from suspensions of ZnO_2_ and ZnO_2_/ppy. Both ZnO_2_ and ZnO_2_/ppy produced significant amount of H_2_O_2_ even in dark. But ZnO_2_/ppy composite showed nearly twice the amount of H_2_O_2_ than ZnO_2_. Figure 4b shows NBT degradation of by superoxide radicals produced from ZnO_2_/ppy in dark. A significant increase in degradation of NBT by ZnO_2_/ppy was observed, indicating enhancement in the production of superoxide radicals compared to ZnO_2_ (Supplementary Fig. S5). Figure 4c,d show the fluorescence spectra of hydroxyl terepthalic acid obtained from ZnO_2_/ppy in dark and in visible light. Hydroxyl radical concentrations were estimated for the nanocomposite both in dark and in visible light. It was observed that the amount of hydroxyl radicals under visible light was greater than the hydroxyl radicals in dark, 2.85 and 1.2 ppm respectively. The kinetics of hydroxyl radical production is shown in supporting information (Supplementary Fig. S6).

### Dye degradation by ZnO_2_/ppy in dark

Figure 4e,f show catalytic degradation of RhB (10 ppm) and MB (5 ppm) by ZnO_2_/ppy in dark. The product degraded both the dyes (100% and 81% respectively) within 20 minutes as indicated by the decrease in absorbance confirming catalytic degradation. Neither ZnO_2_ nor ppy showed degradation of RhB and MB (Supplementary Fig. S7) in dark. It indicates physiochemical synergy between ZnO_2_ and ppy in the nanocomposite that causes degradation of dyes in dark.

### Kinetics of RhB dye degradation in dark

**Figure 5:**
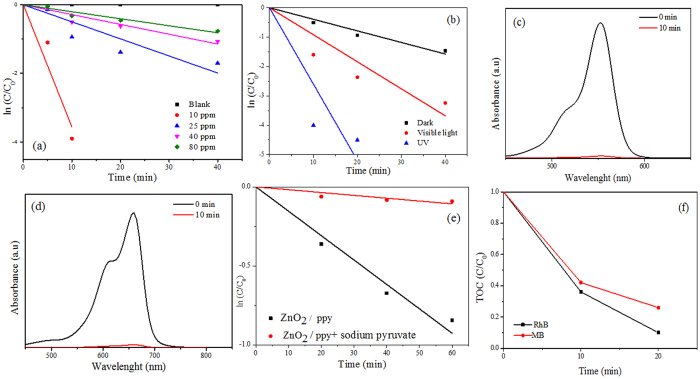
(**a**) Degradation kinetics of various concentrations of RhB by ZnO_2_/ppy in dark (**b**)Degradation kinetics of RhB (25 ppm) based on source of irradiation (**c**) Degradation of RhB (25 ppm) by ZnO_2_ under UV light (**d**) Degradation of MB by ZnO_2_under UV light (**e**) degradation kinetic of RhB (40 ppm) by ZnO_2_/ppy in dark in presence and absence of sodium pyruvate (**f)**Temporal change in TOC during degradation of RhB and MB by ZnO_2_/ppy in dark.

Figure 5a shows the degradation kinetics of different concentrations of RhB with time. The % degradation for RhB of concentrations 10 ppm, 25 ppm, 40 ppm and 80 ppm are 100, 81, 65 and 54 respectively (Supplementary Table S1). The first order rate constant for 10 ppm is 35 × 10^−2^ min^−1^ even under dark (Table 1). Table 1 lists the performance of ZnO_2_/ppy towards RhB degradation. The degradation efficiency of ZnO_2_/ppy is relatively higher than that of the other systems in dark and in light. Degradation of RhB of different concentrations is given in supporting information (Supplementary Fig. S8).

### Degradation of dyes in presence of light

Figure 5b shows degradation kinetics of RhB (25 ppm) by ZnO_2_/ppy under different sources of irradiation. The percentage of degradation under intense visible light was 96 and under UV light, it was 100 (Table 1) within 60 and 10 minutes respectively. The rate of degradation of RhB under UV light is found to be thrice that of visible light (Table 1).

Supplementary Fig. S9 shows the degradation of MB (5 ppm) under intense visible light. Percentage degradation under intense visible light was 92, and under UV it was 100% within 20 minutes and 10 minutes respectively. The rate of degradation of MB under UV light is four times that of visible light (Table 1). Most interestingly, under UV irradiation, ZnO_2_ itself could degrade (100%) both 25 ppm of RhB (Fig. 5c) and 5 ppm of MB within 10 min (Fig. 5d). The effect of light (visible and UV) with respect to % degradation could have been observed at higher concentrations of the dyes.

### Total Organic content (TOC)

In order to confirm the degradation products of RhB and MB by ZnO_2_/ppy, TOC of reaction mixture drawn at regular intervals during dye degradation was analysed. The aqueous suspension of ZnO_2_/ppy in the absence of dye was taken as control. Significant decline in TOC was observed for both dyes within short time (Fig. 5f).

### Photodegradation stability of ZnO_2_/ppy

The synthesized composite is stable even after irradiation with light (UV and Visible). Supplementary Fig. S10 (XRD) confirms the stability of products even after degradation of dye.

### Mechanism of ROS generation and dye degradation under dark

Reactive oxygen species (^·^OH, ^·^O_2_^−^) is considered responsible for photocatalytic activity (resulting in degradation of dyes) of semiconductor materials in suspensions. The nanocomposite under study could degrade dyes through the release of ROS even in dark, whereas neither ZnO_2_ nor ppy alone degrade dyes in dark. This confirmed the symbiotic role of both in degradation of dyes. During the process, ROS was produced, as shown in equations (4, 5, 6, 7, 8, 9, 10). To understand this, a mechanism, hitherto not reported, involving free electrons from ppy is proposed. Polypyrrole is a conductive polymer with extending π-conjugated electron systems^34^. During the oxidation of pyrrole by ZnO_2_ in presence of Cl^−^ ion, electron radical was formed on the carbon site of polypyrole chain, as indicated below^49^,^55^.


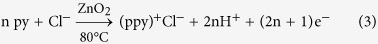


ppy synthesized by oxidative process contain free electrons on the polymer chains even in the absence of light. In the presence of light, more electrons are excited from valence band to conduction band of ppy, increasing the number of free electrons in conduction band and it is supported by conductivity and Electron Spin Resonance (EPR)studies^56^,^57^. ESR of ppy^+^ shows the presence of free electrons (Fig. 3e).

Metal peroxides in water releases H_2_O_2_^58^ (equation 4) and it is also estimated in our study (Fig.4a). The released H_2_O_2_, being a scavenger of electrons, accepts electrons from ppy that is present on the surface of ZnO_2_ (Fig. 3f) forming hydroxyl radical and hydroxyl ion (equation 5).


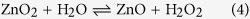



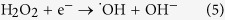



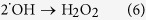



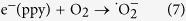



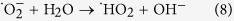



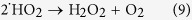



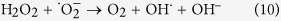


Free electrons from ppy can combine with O_2_ in suspension to form superoxide radical as shown in equation (7), thereby resulting in H_2_O_2_ as in equation (9). Superoxide formation was confirmed by NBT degradation (Fig. 4b) and H_2_O_2_ (Fig. 4a) & OH^·^ (Fig. 4c,d) were also estimated.

ZnO_2_ alone did not produce ^·^O_2_^−^ in aqueous suspension, which points to the significant role of ppy in production of superoxide, as confirmed through equation (7). Hydroxyl radicals produced in equation(5) can recombine to form H_2_O_2_, an additional source other than equation(4). It leads to a greater production of H_2_O_2_ from ZnO_2_/ppy than ZnO_2_ itself, as confirmed through KMnO_4_ titrations (Fig. 4a). Equations (4) - (10) illustrate the catalytic consumption and generation of oxygen.

### Scavenging study of H_2_O_2_

To understand the role of H_2_O_2_ in the generation of ^·^O_2_^−^& OH, which are necessary for the degradation of dyes (RhB), H_2_O_2_ scavenging study using sodium pyruvate, an effective scavenger of H_2_O_2_, was done. 50 mg of sodium pyruvate was added to 40 ppm RhB dye and degradation of dye was monitored. Degradation efficacy decreased from 65% to 8% in the presence of scavenger, which confirmed the significant role of H_2_O_2_ in the degradation of dyes (Fig. 4e). It is to be noted that H_2_O_2_ alone could not degrade RhB. It is well established that the generated superoxide radicals and hydroxyl radicals can degrade the dyes. The above results also prove that ROS produced from ZnO_2_/ppy caused the degradation of RhB and it is not due to adsorption.

### Mechanism of ROS generation in visible light

**Figure 6:**
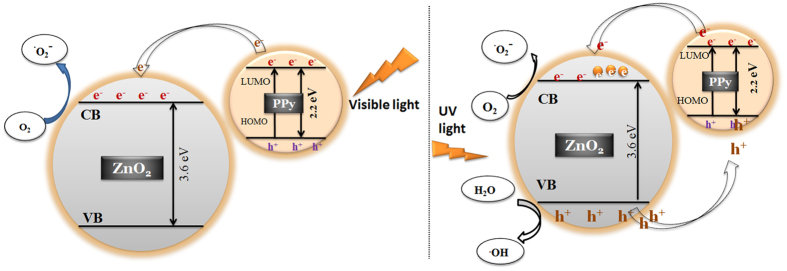
Band structure and mechanism of generation of ROS under visible and UV light.

The band gap of polypyrrole is 2.2 eV^42^ and on irradiation with visible light ppy might act as photosensitizer, injecting electrons to the conduction band of ZnO_2_. These react with dissolved oxygen producing superoxide radicals (equation 11, 12, 13). Superoxide radicals react with water to form ROS, as shown in equation (8, 9, 10). Reduction in band gap was observed in ZnO_2_/ppy composite comparing ZnO_2_ (3.6 eV to 2.86 eV). The valence band (VB) and conduction band positions along with the HOMO (Highest Occupied Molecular Orbital) & LUMO (Lowest Unoccupied Molecular Orbital) of ppy are depicted in Fig. 6. ppy can absorb visible light, thereby causing excitation of electrons from HOMO to LUMO of ppy and the excited electrons from LUMO of ppy get transferred into the conduction band of ZnO_2_ across the interface due to energy match & chemical synergy. These electrons produce ROS (equation 5, 6, 7, 8, 9, 10) that enhance the photocatalytic activity. Electrons from VB of ZnO_2_ can migrate to HOMO of ppy and hence e-h pair is effectively separated (Fig. 6). This mechanism operates in addition to the mechanism that operates in the dark.


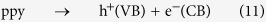



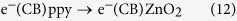



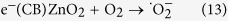


### Mechanism of generation of ROS under UV irradiation

The band gap of ZnO_2_ is 3.6 eV. On exposure to UV light, electrons will be excited to conduction band leaving holes in valence band (Fig. 6). Electrons and holes react with dissolved oxygen and water to form ROS as shown in equations (1, 2, 3, 4, 5, 6, 7) of supplementary information. Under UV light, holes created in VB of ZnO_2_ can migrate to the HOMO of ppy due to energy match and hence this synergy results in effective electron- hole separation increasing the photocatalytic effect. The recyclability of the nanocomposite has also been tested.

## Conclusion

A new nanocomposite ZnO_2_/ppy has been synthesized and characterized. This nanocomposite could degrade the dyes significantly both in dark and light through ROS. Generation of ROS from ZnO_2_/ppy in dark is reported for the first time, and its concentrations have been estimated. The rate constants of dye degradation both in dark and light are found to be higher than those systems reported earlier. Significant enhancement in rate of degradation has been observed on irradiation with visible and UV light. The present work is of much relevance for commercial applications in the degradation of dyes in effluents.

## Methods

### Synthesis of ZnO_2_

2 M of KOH (25 ml) was added to 1 M of Zn(CH_3_COO)_2_ (25 ml) solution at room temperature under stirring. A white precipitate was formed. After 30 min, 30 ml of 30% H_2_O_2_ was added to the precipitate and stirred at room temperature for 1 h. The precipitate was centrifuged at 8000 rpm, pellet washed twice with water and dried in oven at 100 °C^58^.

### Synthesis of ZnO_2_/ppy composite

100 mg of ZnO_2_, 100 μl of pyrrole, 100 μl of 0.14 M of SDS and 10 μl of Con HCl were added to 10 ml water in a closed container, and it was kept in an oven at 80 °C for 4–6 h. The powder was washed with water, centrifuged at 8000 rpm and dried in oven at 80 °C. The composition of the product corresponds py: ZnO_2_ of 10:100 in weight. We also synthesized nano composites in the ratio of 5:100 & 20:100 of py to ZnO_2_. But the activity was found to be maximum for 10:100 ratio, and hence the results presented here pertain only to this ratio.

### Characterization

Phase purity and crystallite size of synthesized products were analyzed by Bruker D8 Advance powder X-Ray Diffractometer (Bruker AXS GmbH, Karlsruhe, Germany) with CuKa source. The morphology and particle size of synthesized products were examined using Transmission Electron Spectroscopy (TEM). TEM has been recorded employing JEOL JEM 3010 electron microscope (JEOL Ltd., Tokyo, Japan). Photoluminescence spectrum (PL) was recorded using Hitachi F-7000 Fluorescence spectrophotometer with 150 W Xe lamp as excitation source. The slit width at excitation and emission were 5 nm. UV-Visible spectra were recorded at room temperature using Jasco V 570 UV-Vis spectrophotometer. X-Ray photoelectron spectroscopic analysis was done using K-Alpha instrument (XPS K-Alpha surface analysis, Thermo fisher scientific, UK). X-band EPR was recorded using Varian E 112 at room temperature. Total Organic Content (TOC) was measured using Shimadzu TOC-L.

### Estimation of H_2_O_2_

H_2_O_2_ generated from aqueous ZnO_2_and ZnO_2_/ppy suspensions was estimated by KMnO_4_ redox titrations^58^. To aqueous suspensions of ZnO_2_ and ZnO_2_/ppy, 2 ml each of KMnO_4_ and H_2_SO_4_ were added at appropriate concentrations, kept under constant stirring at room temperature under ambient light and in dark. At regular intervals, 5 ml aliquots were filtered through membrane filter. H_2_O_2_ was estimated by standard titrations.

### Estimation of Hydroxyl radical (^·^OH)

Hydroxyl radicals were estimated using fluorescence spectroscopy. Terepthalic acid (TA) with hydroxyl radicals forms 2- hydroxyl terepthalic acid complex which gives fluorescence and its intensity is a direct measure of hydroxyl radical concentration^44^. In a typical procedure, to aqueous suspensions of ZnO_2_ and ZnO_2_/ppy, 2 mM of TA were added and stirred under ambient light. At regular intervals, 2 ml aliquots were withdrawn, filtered through membrane filter, and the fluorescence was measured at excitation wavelength at 312 nm. The intensity of emission at 425 nm was correlated to hydroxyl radical concentration.

### Estimation of superoxide (^·^O_2_^−^)

Superoxide radicals from aqueous suspensions of ZnO_2_and ZnO_2_/ppy were estimated by Nitro blue Tetrazolium (NBT)^59^. NBT showed maximum absorbance at 259 nm but with superoxide radicals, it was converted to mono formazon and difarmozon. The production of superoxide radicals was estimated by monitoring the degradation of NBT using UV-Visible Spectroscopy.

### Dye degradation by ZnO_2_ and ZnO_2_/ppy

40 mg of catalyst was added to 25 ml of RhB (20 ppm)/25 ml of MB (5 ppm) under stirring. At regular intervals, 2 ml of the solution was taken, centrifuged at 8000 rpm and UV vis spectroscopy was recorded for supernatant. All the experiments were conducted in darkness, in visible light and under UV irradiation. 300 W halogen lamp with spectral distribution form 350–2000 nm was used for visible irradiation. Photocatalytic dye degradation under UV was done using Heber multi lamp photoreactor with 8 W mercury lamp at wavelength of 254 nm.

## Additional Information

**How to cite this article:** V., L. P. and Rajagopalan, V. A New Synergetic Nanocomposite for Dye Degradation in Dark and Light. *Sci. Rep.* **6**, 38606; doi: 10.1038/srep38606 (2016).

**Publisher's note:** Springer Nature remains neutral with regard to jurisdictional claims in published maps and institutional affiliations.

## Supplementary Material

Supplementary Information

## Figures and Tables

**Table 1:** Comparison of performance of different materials towards dye degradation.

Composition	Dye	Concentration of dye (ppm)	Dark		Visible		UV	
			%	k (min-1) × 10^−2^	%	k (min-1) ×10^−2^	%	k (min-1) ×10^−2^
ZnO_2_/ppy	RhB	10	100	35.6	N.S	N.S	N.S	N.S
ZnO_2_	RhB	25	0	0	0	0	100	18
ZnO_2_/ppy	RhB	25	81	4.9	96	9.1	100	26
ZnO_2_	MB	5	0	0	0	0	100	32
ZnO_2_/ppy	MB	5	83	4	95	8.9	100	35
Degussa TiO_2_^50^	RhB	10	—	—	N.S	N.S	100	28
TiO_2_^50^	RhB	10	—	—	N.S	N.S	100	1.4
TiO_2_/ppy^51^	MB	10	—	—	100	3.4	N.S	N.S
TiO_2_/PANI^52^	RhB	10	—	—	100	3.39	100	11.3
ZnO/PANI^53^	MB	10	—	—	85	0.41	10	6.6
ZnO^54^	RhB	10	—	—	N.S	N.S	7.5	100

N.S.- not studied.
