# Supplementary material for: A New Synergetic Nanocomposite for Dye Degradation in Dark and Light
Source: Sci Rep. 2016 Dec 8;6:38606. doi: 10.1038/srep38606 (PMC5144144; doi:10.1038/srep38606)
Supplement: Supplementary Information [file srep38606-s1.pdf]

A New Synergetic Nanocomposite for Dye Degradation in Dark and Light

V. Lakshmi Prasanna & Rajagopalan Vijayaraghavan*

Department of Chemistry, School of Advanced Sciences, VIT University, 632 014,Vellore, India

Author contact information:

*E-mail ID : rvijayaraghavan@vit.ac.in

Ph : +91 416 220 2322

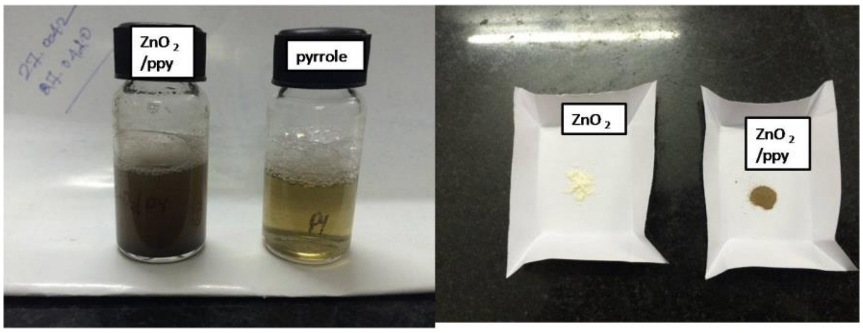

Figure S1: colour of ZnO_2 / ppy composite aqueous suspension after reaction and colour of ZnO_2 and ZnO_2 / ppy powder after cebrifufgation

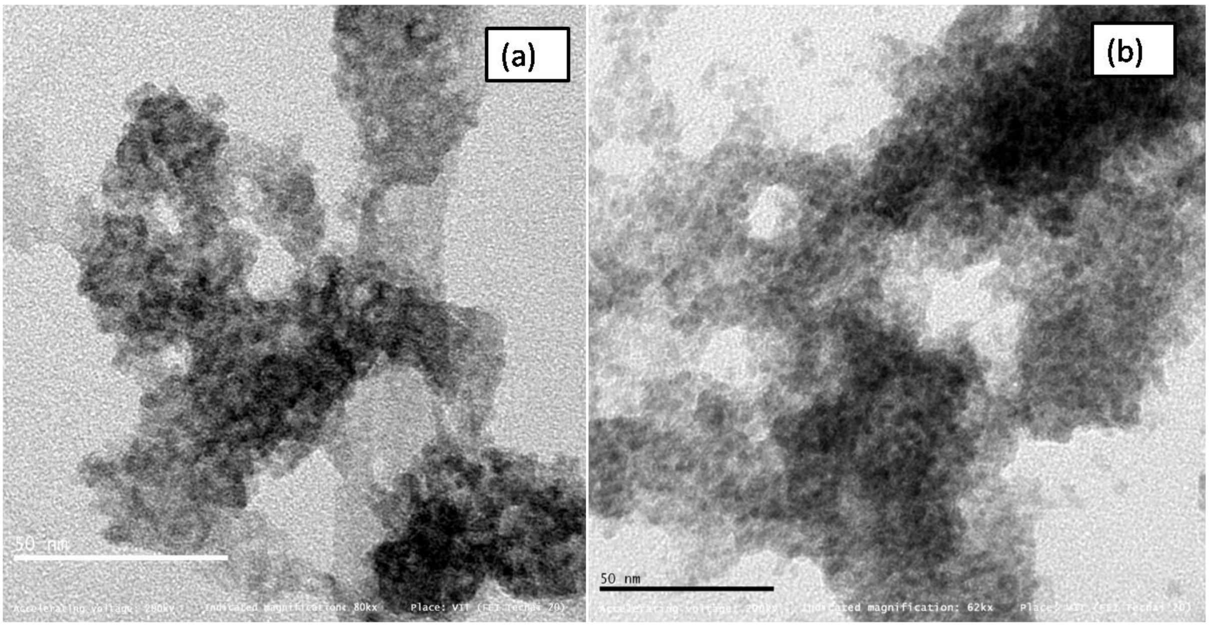

Figure S2: TEM of (a) ZnO_2 (b) ZnO_2 / ppy

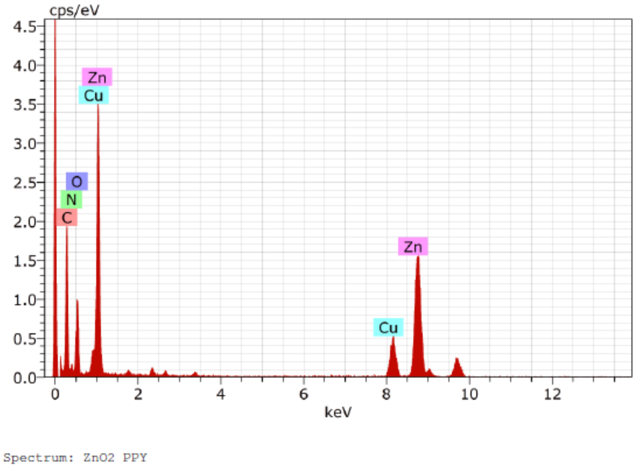

Figure S3: EDAX of ZnO_2 / ppy

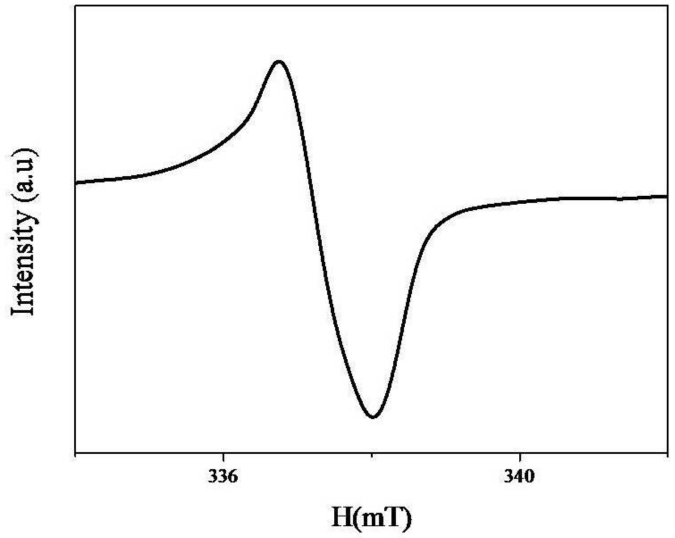

Figure S4: EPR of ZnO_2

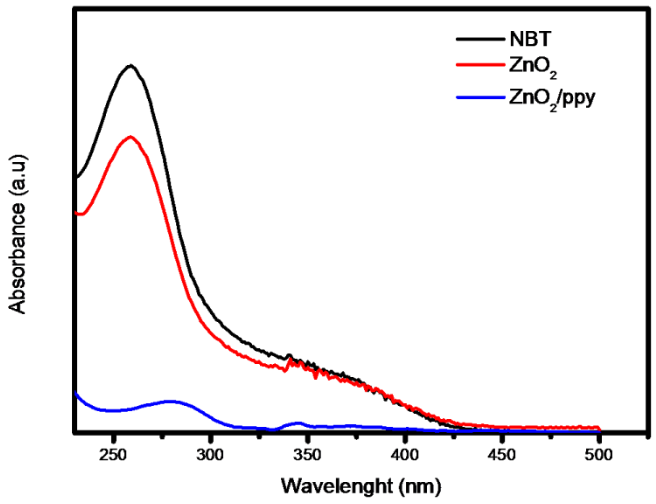

Figure S5: Degradation of NBT by ZnO_2 and ZnO_2 / ppy after 2h

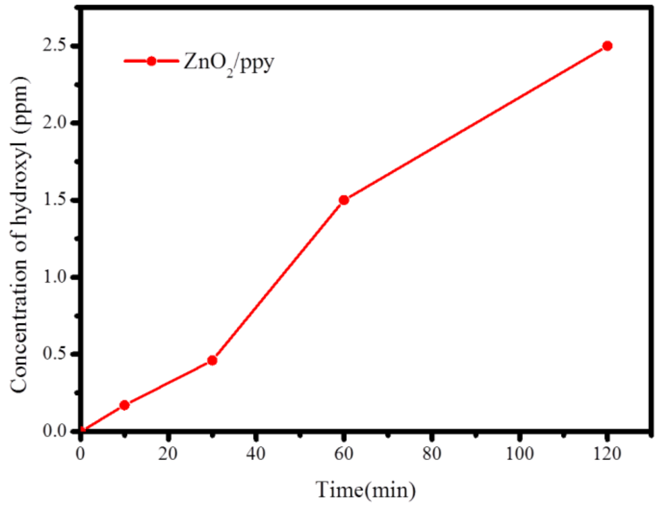

Figure S6: Kinetics of hydroxyl production in dark

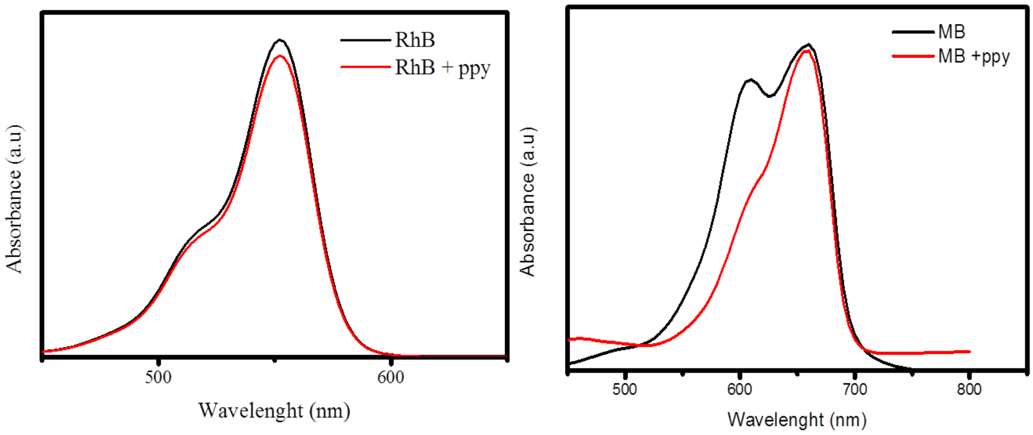

Figure S7: UV vis spectroscopy of RhB and MB in the presence of polypyrrole

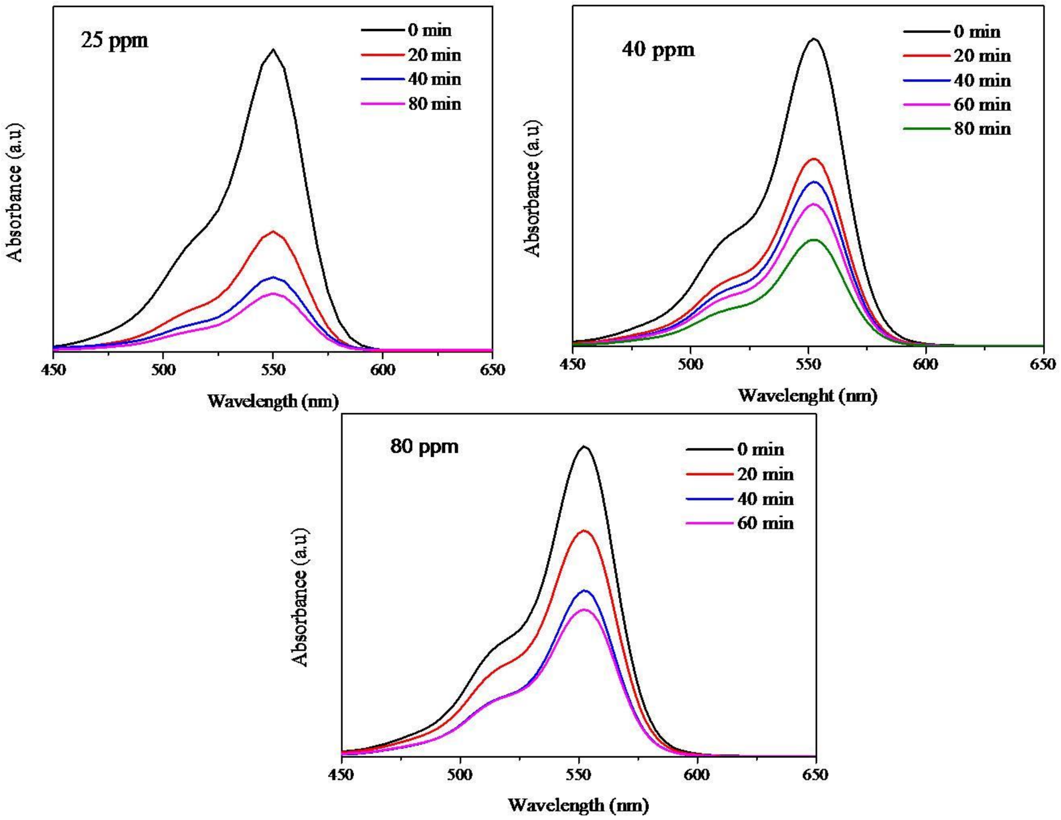

Figure S8: Degradation of RhB by ZnO_2 / ppy at different concentration

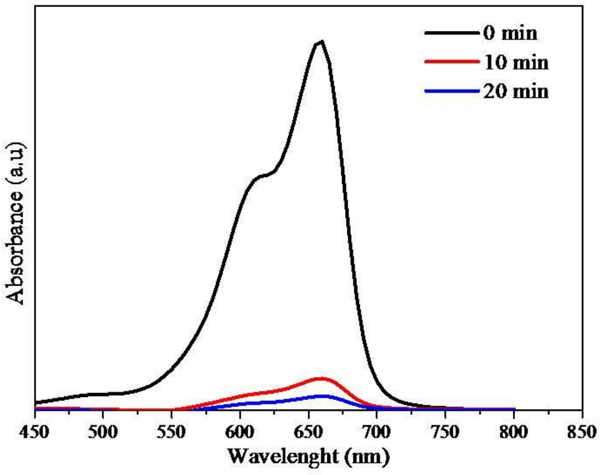

Figure S9: Degradation of MB (5 ppm) by ZnO_2 / ppy under visible light

Table S1: kinetics of degradation of RhB with concentration of ZnO_2 / ppy in dark

Concentration of dye (ppm)	% of degradation	k(min^{-1}) X 10^{-2}
10	100	35.6
25	81	4.9
40	65	2.8
80	54	2.0

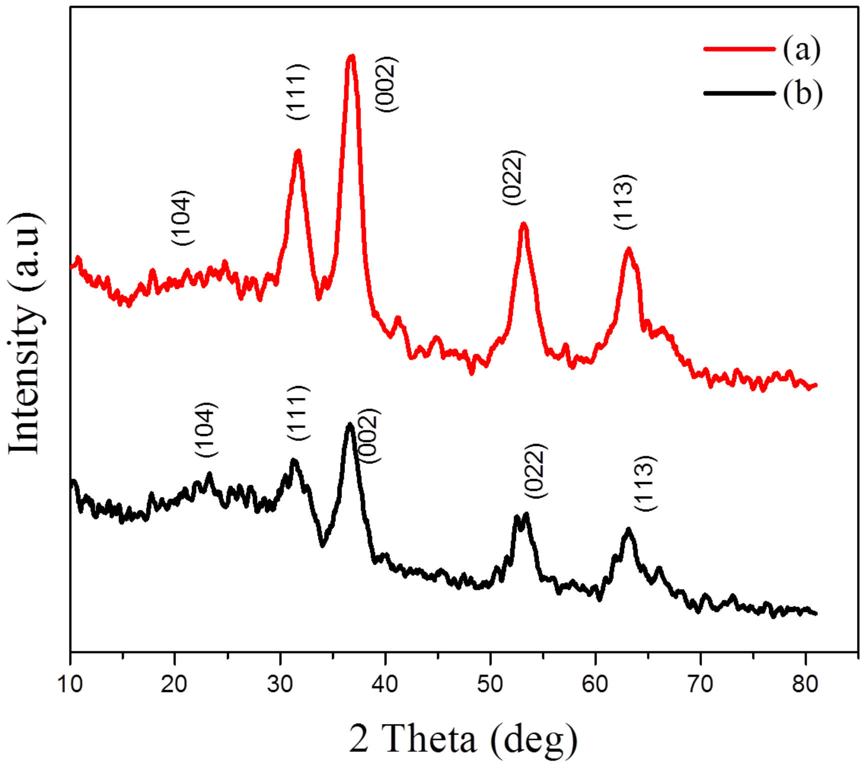

Fig. S10: XRD patterns of ZnO_2 / ppy after dye degradation in (a) Dark (b) visible light

$$ZnO_2 + h\upsilon \longrightarrow e^- + h^+ \quad (1)$$

$$H_2O + h^+ \longrightarrow {}^{\cdot}OH + H^+ \quad (2)$$

$$O_2 + e^- \longrightarrow {}^{\cdot}O_2^- \quad (3)$$

$${}^{\cdot}O_2^- + h^+ \longrightarrow {}^{1}O_2 \quad (4)$$

$${}^{\cdot}O_2^- + H^+ \longrightarrow {}^{\cdot}HO_2 \quad (5)$$

$${}^{\cdot}HO_2 + H^+ + e^- \longrightarrow H_2O_2 \quad (6)$$

$${}^{\cdot}OH + {}^{\cdot}OH \longrightarrow H_2O_2 \quad (7)$$

Scheme S1 : Mechanism of ROS generation under UV irradiation
